# Mini research projects as a mechanism to improve the quality of dementia care

**DOI:** 10.1186/s13584-018-0273-5

**Published:** 2019-02-20

**Authors:** Hava Golander

**Affiliations:** Department of Nursing and Herczeg Institute for the Study of Aging and Old Age, Tel Aviv University, Tel Aviv, Israel

**Keywords:** Dementia methodology, Psychogeriatric care policy, Quality of care, Service-academia collaboration

## Abstract

Several models have been proposed to connect academia and practice in order to improve long-term care. In this paper we propose and describe the “Mini-Research Group” as an alternative model of such collaboration. The formation of mini-research groups was the unplanned by-product of a longitudinal action research project headed by the late Prof. Rebecca Bergman, a prominent nursing leader from the Department of Nursing at Tel-Aviv University. It involved a two-stage project aimed at developing, and later implementing, a specific tool to evaluate the quality of care provided in geropsychiatric units and to design a nursing intervention which entailed an improved model for care in specialized geropsychiatric units for persons with dementia. Initially, this article describes the projects that led to the development of mini-research groups, and then continues to describe several mini-research projects, focusing on the research questions which emerged from practice as well as the variety of methodologies used. Finally, we discuss the ways in which mini- research groups contributed to the quality of care for persons with dementia, benefited their families, professional staff, faculty participants, and advanced policy development. We argue that in light of the present array of ethical and legal restrictions which inhibit the recruitment of participants, using mini-research groups combined of practitioners and researchers, can provide a pragmatic solution, not only to overcome these barriers, but to improve the quality of care, stimulate clinical dementia research, and promote new insights into the lives of persons with dementia.

## Background

Several models have been proposed to connect research with practice in order to improve long-term care, among them research institutes affiliated with nursing homes, clinician-initiated research programs, or the more comprehensive tri-focal model of care which combines patient centered care, positive work environment, and evidence-based practice under one big umbrella which fosters a collaborative relationship between nursing homes and academic institutions. [1–3] Despite their prior successes, these models seem to have disappeared from the field of dementia research. This paper sets forth an Israeli model for addressing this and other challenges: the mini-research group.

The initiative to improve and evaluate the effectiveness of care in geropsychiatric units, which was started by Prof. Rebecca Bergman in 1985 and completed in 1992, produced important lessons for understanding persons in advanced stages of dementia and for assessing care provided and research conducted in geropsychiatric units. This longitudinal action research involved about 70 nurses from 20 geriatric centers in Israel, national geriatric inspectors from the Ministry of Health and faculty members from Tel Aviv University. The establishment of the mini-research groups was one of a number of unplanned positive outcomes which emerged from this project [4].

**Fig. 1:**
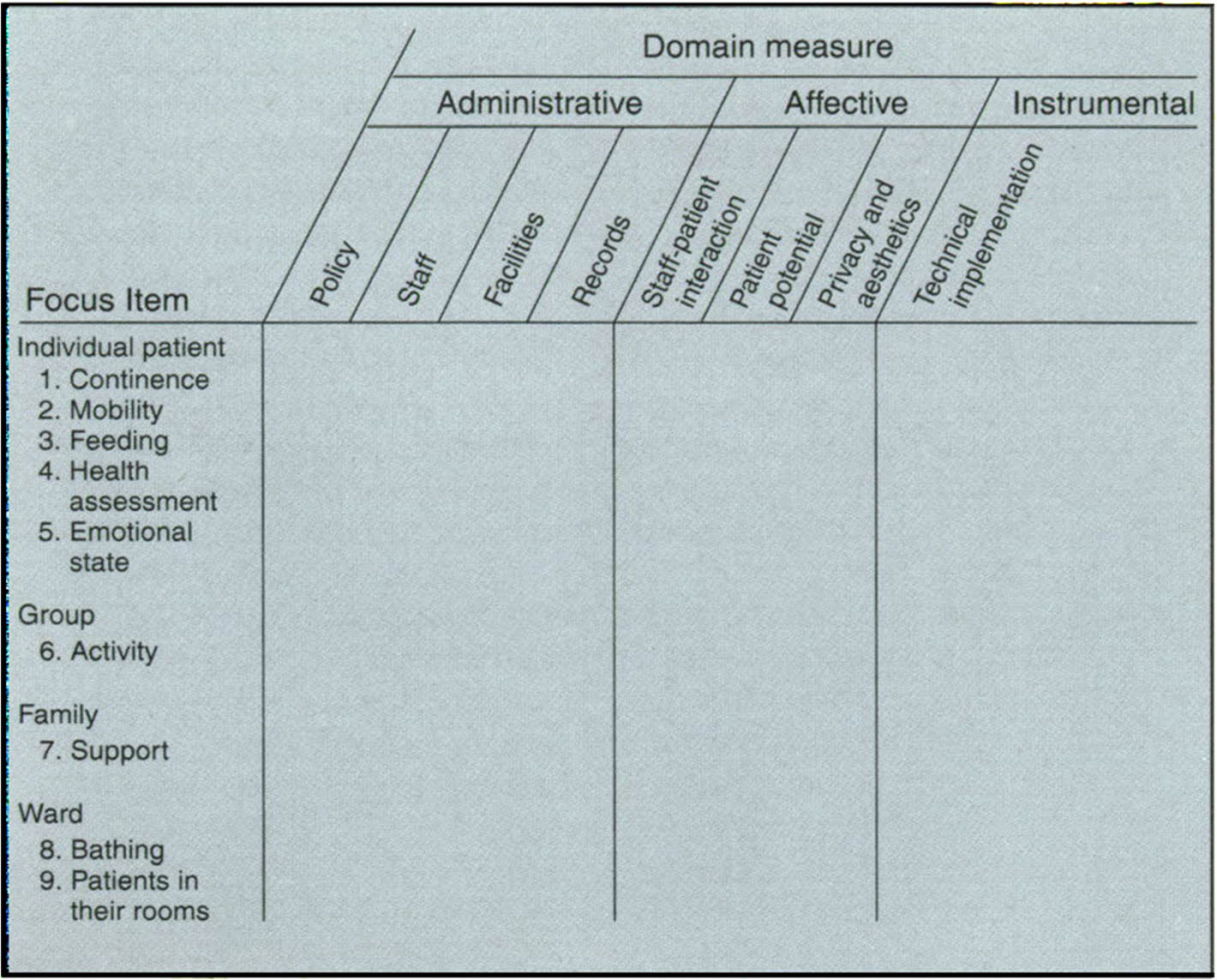
A model of the two-dimensional model containing 72 cells

A previous report of an interdisciplinary committee on “quality of care in services for the elderly” [5] provided a comprehensive framework for Prof. Bergman’s project. The basic undifferentiated model consisted of six major domains: physical environment, psychological environment, basic personal care, health care, family involvement and human resources. Thus, the first stage of the project involved further developing a specific model which would be relevant and unique to the geropsychiatric units’ characteristics. This involved reviewing the literature, conducting on-site observations, and interviewing residents, families and staff caregivers. The tool that was developed was tested in several settings [6]. It related to residents as individuals, as groups, and to the unit as a whole. The model provided for 72 cells which evaluated nine focus items on eight administrative, affective, and instrumental measures, as shown in Fig. 1.

The second phase of the project included the implementation of the tool. The leading project team organized bimonthly full day meetings attended by more than 70 nurses, including unit nurses and directors from 20 geriatric centers, national geriatric nursing inspectors, and nursing faculty from Tel-Aviv University. Each gathering, hosted by a different geriatric center, followed a similar format: presentation of a background paper, discussion of one of the measures of care, guided tours of geropsychiatric units, and exchanges of information regarding problems and experiences related to the topic in discussion. In addition, the project core team, consisting of three geriatric nurse specialists, provided in depth guidance to six non-profit geropsychiatric units during weekly site visits. The team focused on identifying needs, planning and implementing change, and encouraging grass-roots involvement in every phase of the process. One year later, a follow up study showed improved quality of care, retention of positive changes and higher satisfaction among residents, families and staff as compared to the status quo at the project’s onset [7].

The mini-research groups, an outgrowth of the project’s large group meetings, continued to operate far beyond the official termination of the project (about 10 years). Each group consisted of practical unit nurses, guided by an academic advisor, and focused on a common unresolved clinical problem, which was raised by the clinical staff. With the help of the academic advisors, the problems were framed in terms of systematic research questions, with the goal of formulating appropriate interventions for challenging issues. Favorable results from one study group encouraged the establishment of additional mini-groups to solve other problems within the psychogeriatric unit’s daily routine. Altogether, about 15 mini-research groups were convened to study a wide range of clinical problems, such as how to use Jacuzzi bathing as a therapeutic tool; how to address loneliness; and how to reduce violence.

In order to illustrate how the insights gained from a mini-research project can serve to promote the understanding of dementia and the improvement of care, several exemplars of successful mini-research projects are presented herein, each with its distinctive incentive, methodology and outcomes.

### Examples of the mini-research projects

- *The “Violence Group” Reducing violence among geropsychiatric residents:*

Violent outbursts by residents are common occurrences in geropsychiatric settings. The study team decided to study what triggers outbursts of violence. What cues in the resident’s behavior might indicate a mood change? How should violence be categorized? Which interventions can be helpful?

**Fig. 2:**
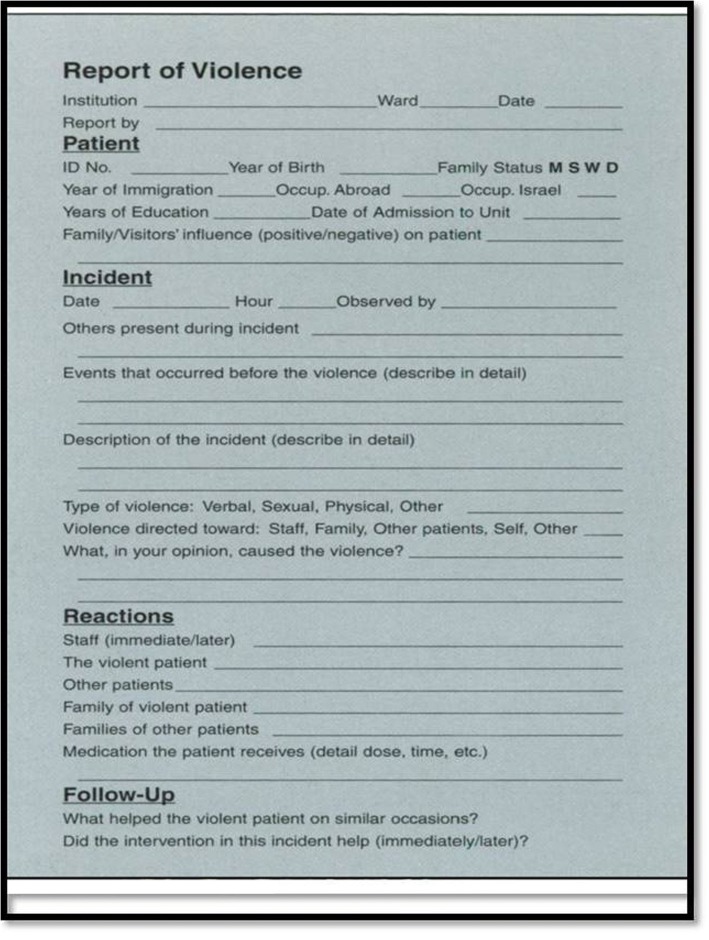
Sample incident report

The group carried out a literature review, gathered more than 30 observed and reported relevant incidents, documented them on a semi-structured questionnaire which they developed (see Fig. 2) and, using qualitative techniques, analyzed the data in relation to the residents’ characteristics, the nature of the violent act, the reactions of others, and which interventions were effective. The study team presented its findings to the greater group and its findings encouraged others to establish additional mini-research groups [4].

b. *The “Mirrors Group” – The use of mirrors as a therapeutic tool for raising self-awareness:*

An occasional observation reported by a nurse about a resident in the geropsychiatric unit ,who was searching obsessively in front of and behind the mirror - provided the incentive to establish another group to examine the effects of mirrors on persons with dementia. How do persons with dementia relate to their image in the mirror? Is the use of mirrors effective in raising levels of self-awareness, calmness and satisfaction? In order to answer these questions, the mini-research group carried out a simple experiment in which 100 persons with dementia were exposed to mirrors of different sizes. Their reactions were documented and analyzed, showing varied responses to looking in the mirror. Most responses were positive (52%) with increased self-awareness regarding personal care, while others were indifferent (10%), or even angered (12%). A majority of residents appeared to benefit from looking at the mirrors. In some instances, the use of mirrors led to improved communication between residents and professional staff. The results of the study team’s work brought to light a new and inexpensive therapeutic tool for persons with dementia: mirrors [8].

c. *The “Dolls Project” – The use of dolls as a therapeutic tool to awaken pleasurable affective responses:*

The therapeutic use of dolls in dementia, though still controversial, is becoming more prevalent at nursing homes and dementia centers. Supporters say that dolls can lessen distress, improve communication and reduce the need for psychotropic medication. Critics say that dolls are demeaning and infantilize seniors. The Dolls mini-research project was a pioneering attempt to systematically examine the influence of dolls as a sensory stimulus to residents in geropsychiatric units [9]. Using a simple experimental design, the staff placed a variety of human and animal figures in a central location inside the activity rooms of 5 units. Using a pre-coded form, the staff observed reactions to the presence of the dolls, method of selection, type of contact, verbal and body communication, behavior of family members and others, and the emotional impact of the dolls. While the attention span of the residents to the dolls varied from a few moments to several hours, the data revealed that more than half of the 100 residents appeared to be happy with the dolls. The residents usually selected “their” same doll. Touching or holding the dolls elicited pleasure, reassurance, and comfort, often stimulating nonverbal communication, with the potential for verbal communication and better interaction between residents and staff. Thus, the researchers found that dolls can be used therapeutically to awaken pleasurable affective responses in persons with dementia.

d. *The “Jacuzzi Bath Project”* – The use of Jacuzzis as a therapeutic tool to address the needs of specific residents.

The Jacuzzi research group was actually formed in order to solve a space-management problem: The luxurious Jacuzzi room in one of the units was reduced to a storeroom because the staff was concerned that entering the tub or bathing might cause residents to feel anxiety, confusion, or might provoke them to violence. The group decided to study whether the Jacuzzi could be used therapeutically. A review of the literature did not produce any relevant information, although hydrotherapy is widely accepted. The methodology incorporated a series of case study analyses. The unit team was encouraged to identify residents whose specific problems might be ameliorated through use of the Jacuzzi. The staff provided an inviting Jacuzzi experience and later evaluated the impact of the treatment in selected situations: a person with aggressive behavior; two night wanderers; and a woman with severe body pain due to arthritis. All the Jacuzzi baths produced a beneficial effect, and the staff overcame their concerns about possible harm to the residents. Consequently, two additional nursing homes participating in the project decided to place Jacuzzis in their geropsychiatric units.

e. *The “Social Networks Project” – Understanding the interpersonal relationships among residents in a geropsychiatric unit.*

**Fig. 3:**
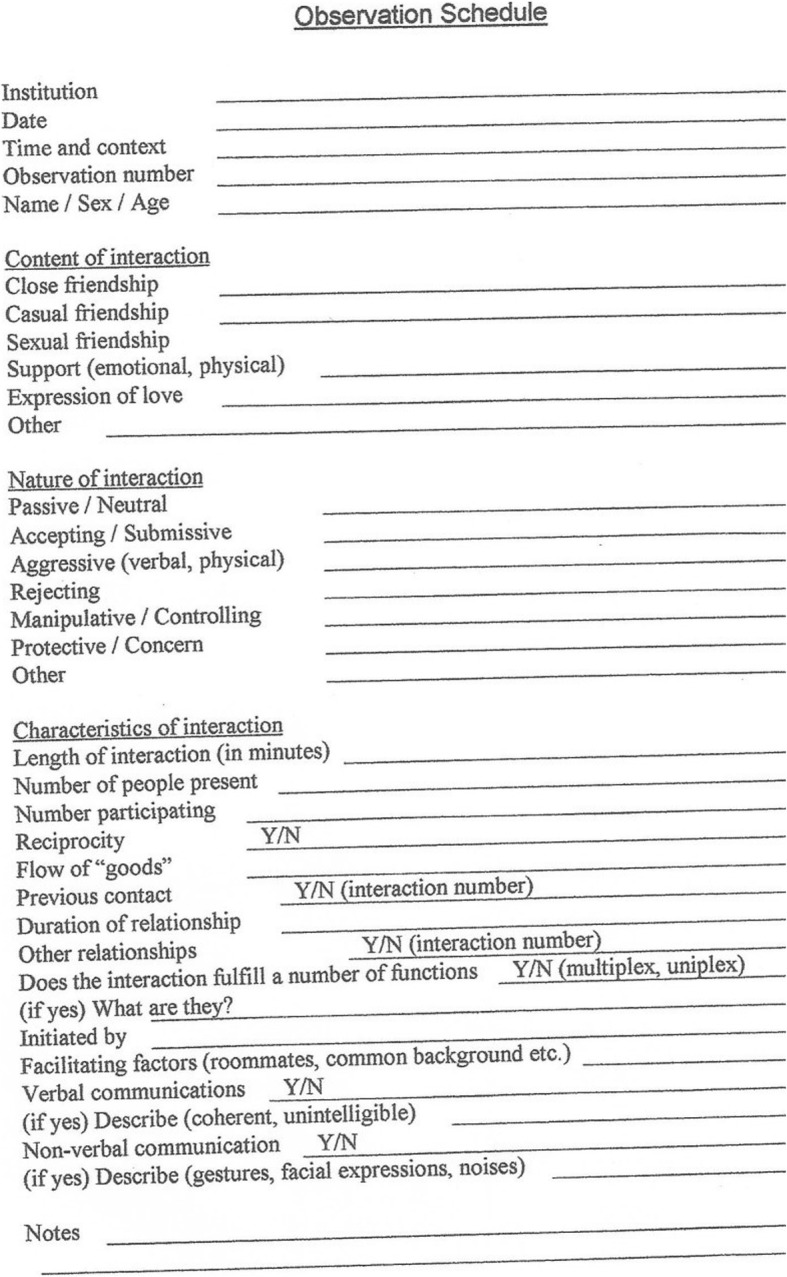
Obsevation Schedules Socialities

Several nurses were interested in examining the potential for establishing social networks among residents with dementia and the possible impact on the residents’ quality of life. The nurses wished to see if altering the social environment could enhance relationships. This project later developed into a research thesis conducted by graduate student Perri Cohen [10]. The methodology chosen was a semi-structured open questionnaire (see Fig. 3 observation schedule). It included the description of a relationship, the morphology of each tie (dyad, triad, or cluster structure), the psycho-social nature of the tie (aggressive/passive/friendly), the degree of symmetry in engagement, the function of the tie (intimacy/being together/help/control etc.) and the identification of the initiator. The depth of the tie and the relationship of the environment to the tie were also observed. Data analysis incorporated qualitative and quantitative methods. The results showed that 44% of the residents with dementia were involved in a consistent social tie of some kind, most often observed as “being together” in a dyad (80%), or in a “concern and help” relationship (66%). The resident’s background variables did not influence the formation of social ties, and neither did his/her cognitive or physical function. Significantly, most of the ties were developed between two residents with different levels of function. This seemed to allow for reciprocity and for the enhancement of self-esteem for both parties. The study concluded that social skills, preferences and abilities were relatively preserved in residents with dementia even for those in the more advanced stages of the disease, suggesting that staff members can play a more active role in facilitating the social environment of the residents than previously thought. For example, staff members can maintain a resident’s grooming and aesthetic appearance to promote social interaction, and can promote a friendly atmosphere in the unit for the overall well-being of residents.

### Contributions of the mini-research group

The impact of the mini-research groups was multi-dimensional and relatively long lasting. The four major contributions of the project were:

- **Improved quality of care -** The mini-research projects had a marked effect on the quality of care in the psychogeriatric units. Staff became more sensitive, attentive and knowledgeable to residents’ potentials and needs. Care became more holistic in the sense of integrating physical, psychological, and social aspects. Nursing interventions tended to become more active, creative, evidence-based, and individualized, compared to the regimented care provided prior to the project.
- **Increased family involvement** - Due to the active role family members played during the project by providing data and feedback to the staff, they became more involved in the unit, They intensified their participation in unit social activities, became closer with the staff and gained an increased general awareness of the needs and potential of their relative and the staff.
- **Improved self-image of nursing personnel -** Personnel employed in the geropsychiatric units traditionally perceive themselves and others employed at nursing homes as holding the least desirable positions in the work world. Those with the opportunity to advance usually preferred more prestigious work environments than those found at geriatric centers. As a result, nursing home staff included few nurses with academic or post-basic preparation. The geropsychiatric project brought a positive change to the self-image of staff members employed in units which participated in mini-research groups. Such staff members became the center of professional attention and the envy of their colleagues in other geriatric units. The geropsychiatric nurses reported that they felt stimulated and challenged and were more eager to continue in their place of work, an environment which had become exciting and rewarding. They felt that they had become more independent in their practice and more knowledgeable, individually and as a group. They took pride in their new practice, they often documented their projects in video and presented their experiences in professional conferences. Upon termination of the formal project, group members decided to continue on their own. They established a national geropsychiatric nurses association, published their own professional journal “The Forum,” organized their own annual conferences, and with some modifications, continue to function as a strong specialty group organization to this day.
- **The merit of collaboration between practitioners and researchers –** The frequent meetings of the mini-research groups provided a model for collaboration between academia and providers that enriched both parties and enhanced mini-research group outcomes. The merit of the collaboration for the practicing nurses seemed most obvious. With guidance by an experienced researcher from academia, staff was introduced to new ways of thinking and developed a research approach to their everyday practice. They learned how to identify problems, focus on goals, review literature, gather data, analyze data, and reach conclusions. An academic advisor, acted as a role model and a facilitator to energize the nurses’ potential individually and as a group.

The merit of such collaboration for the academic research advisors, while less obvious, also warrants favorable comment. The close exposure of the researcher to practicing nurses and to daily life in the clinical field provided him/her with new, enriched and grounded perspectives which assured the relevancy and accuracy of research in relation to the reality experienced by subjects. Through the joint experience of collaboration, the dialogue between practitioners and researchers fostered new ways of thinking, mutual learning and appreciation between clinical practice and academia. The researchers learned to frame and prioritize research questions consistent with the questions’ importance to persons with dementia, staff and family members. They found that “small and simple” research questions were at times more helpful than “complicated and sophisticated” ones. The staff proved that with little guidance, they could become astute and creative partners in collecting and analyzing data generated by observations and other qualitative method techniques, so relevant for the study of dementia, yet so complicated to implement [11]. Collaborating with service personnel also afforded researchers the satisfaction of witnessing the immediate implementation of their research ideas and recommendations.. The combination of direct input and real-life problems in the field, aided by the experience of practitioners and the knowledge of academia researchers proved to be a happier marriage than the hopeful parties could have imagined during their courtship.

## Conclusion

What can we learn today from the experiences of the mini-research groups which operated in the past?, and how can we apply the lessons learned to the future? Effective research and treatment of dementia, and improving the quality of life and promoting social inclusion of persons with dementia have been identified as a global public health priority by the World Health Organization [12]. Yet conducting research into these matters presents complex ethical and methodological issues [13, 14]. For example, while obtaining an Advance Research Directive (ARD) is still considered a valid consent in the first stages of dementia, ethics review committees are often reluctant to permit even qualitative methods studies to be conducted on people in advanced stages of dementia. These and other obstacles hamper progress [11]. The mini-research model is one way of addressing the numerous ethical and legal requirements which hinder advancement. The mini-research group format provides a pragmatic solution, not only in overcoming procedural barriers, but also in stimulating more research and promoting a greater understanding of persons with dementia. The idea of bringing practitioners and researchers together to study and resolve specific issues which arise in clinical settings has innumerable advantages: The model is simple to administer and overcomes bureaucratic and logistical barriers. Mini-research groups can bring about significant and immediate impacts on the quality of care because they examine and work to resolve “real” problems in specific settings. A diverse research team has a greater likelihood to understand persons with dementia. The diversity within mini-research groups increases the likelihood of finding creative paths forward and furthering the professional growth of participating field practitioners and academic researchers, all to the benefit of persons with dementia.

Prof. Bergman started her project with modest funding, but overflowing personal magnetism, enthusiasm, motivation as well as receptivity by the clinical community. In the ensuing years, long term care facilities have become more overwhelmed with clinical, ethical, legal and financial constraints. If improving the care of persons with dementia is indeed a global goal, achieving progress will require not only sufficient resources and infrastructure, but the selection of effective models for advancing knowledge and implementing best practices. The collaboration of practitioners and researchers in mini-research groups can provide an answer to many of the challenges of addressing the needs of persons with dementia. Yet, to ensure such cooperation on a national level and for long lasting periods, every care policy program should develop and assimilate an appropriate research strategy aimed to increase the knowledge and understanding as well as to ensure the provision of quality care for people with dementia and their family members.
